# Editorial: Immunoparasitology: A Unique Interplay Between Host and Pathogen

**DOI:** 10.3389/fimmu.2020.00880

**Published:** 2020-05-07

**Authors:** Xun Suo, Zhiguang Wu, Hyun Lillehoj, Wenbin Tuo

**Affiliations:** ^1^National Animal Protozoa Laboratory & College of Veterinary Medicine, China Agricultural University, Beijing, China; ^2^Key Laboratory of Animal Epidemiology of the Ministry of Agriculture, China Agricultural University, Beijing, China; ^3^The Roslin Institute, University of Edinburgh, Easter Bush, Midlothian, United Kingdom; ^4^Animal Biosciences and Biotechnology Laboratory, Agricultural Research Service, United States Department of Agriculture (USDA), Beltsville, MD, United States; ^5^Animal Parasitic Diseases Laboratory, Agricultural Research Service, United States Department of Agriculture (USDA), Beltsville, MD, United States

**Keywords:** parasite, helminth, protozoa, Apicomplexa, flagellate, co-infection, immunity, vaccines

This Research Topic, “Immunoparasitology: A unique interplay between host and pathogen,” was intended to emphasize broadly the latest advances in immunoparasitology and has been concluded with more than 30 high quality papers encompassing aspects of parasite biology, host-parasite responses and interactions, and up-to-date control measures. Such an accomplishment is unattainable without the enthusiastic involvement of all contributing authors and participating reviewers, and the day-to-day assistance from the staff of Frontiers in Immunology editorial office.

Approximately one third of the articles in this collection are reviews and the rest are original research papers, covering multiple parasitic species and their hosts. As expected, two thirds of the papers are focused on protozoan parasites, 50% of which use *Toxoplasma gondii* as a model pathogen. The next-most covered group is parasitic helminths with eight papers. At the time this Editorial was submitted for publication, this Research Topic achieved well over 75,000 online views, with average views per article of >2,000 since publication.

The most viewed article covers the popular and important subject of co-infection (Mabbott). The author emphasizes the fact that co-infection is a common occurrence in the field, where malarial parasites, soil-transmitted helminths, bacteria and viruses, are the causes for chronic infections in a large proportion of the population. Ample examples of co-infection are described, where existing infections by parasites can have a dramatic influence on host susceptibility and/or disease pathogenesis. The impact of co-infection on disease diagnosis, vaccine development, and host resistance, clearly warrants further investigation. The findings of Djokic et al. demonstrate specifically how co-infection by *Babesia microti* and *Borrelia burgdorferi*, both tick-borne pathogens, influences age-dependent immune responses and disease outcomes. Additional review articles cover protozoa and helminths, representing up-to-date advances in research. Maurya et al. present an excellent review on the functions of leptin in infectious diseases. Leptin, primarily secreted by adipose tissue, is important in resistance to diseases as it is emerging as a key regulator in both immunity and nutrition.

While malaria and trypanosomiasis are not extensively covered in this issue, in terms of original research, research progress for these two diseases is nonetheless summarized by several in-depth reviews. Lee et al. give an expert account of recent advances in the complex cytoadhesive interactions between *Plasmodium falciparum-*infected erythrocytes and other host cells, providing insight into how such cytoadherence may contribute to malaria pathogenesis. In malaria, B cell-mediated, parasite-specific, antibody-dependent protection may depend on, and be achieved by, appropriate activation in the lymph nodes and optimal interactions among antigen-specific and antibody-secreting B cells with T helper cells and cytokines. This process is described in depth in a review article written by Silveira et al..

Several major species of trypanosomes are the pathogenic agents of trypanosomiasis worldwide, profoundly affecting the well-being and immunocompetency of both humans and animals. To date, there is no vaccine against trypanosomiasis, although the parasite can elicit strong immune responses in the host. The difficulty in developing protective vaccines stems from the ability of parasites to evolve and evade host immune surveillance by genetic exchange among the parasites, acquisition of resistant genotypes in the insect vector, and plasticity in adaptation in new environments (Radwanska et al.). One of the approaches to understanding differential host responses is by investigating routes of transmission. Berreto de Albuquerque et al. summarize their recent findings and those of others in comparing host responses and disease outcomes following *Trypanosoma cruzi* infection through oral or gastrointestinal routes. In Latin American countries, oral transmission of resultant Chagas disease is predominant. In animal models, the oral route of infection can give rise to higher rates of mortality and morbidity when compared to vector-mediated transmission. These authors indicate that oral transmission, in fact, consists of intraoral cavity and intragastric transmissions, with increased parasitemia and mortality through the intraoral cavity route (Berreto de Albuquerque et al.). *T. cruzi* appears to enhance its replication in macrophages by exploiting the cell's Wnt signaling system based upon the ability of Wnt/β-Catenin signaling inhibition *in vitro* and *in vivo* to restrict *T. cruzi* replication, thereby promoting host resistance (Volpini et al.). Similarly, *Leishmania amazonensis*, a close relative of the trypanosome, also promotes its own infectivity using a host cell protein, CD100, through a macrophage surface receptor, CD72 (Galuppo et al.). *Histomonas meleagridis* is a flagellated protozoan parasite, causing histomonosis in turkeys and chickens, with the latter being more resistant to infection (Mitra et al.). Recent studies by Kidane et al. show that, unlike turkeys, resistance in chickens relies on the presence of higher percentage of interferon-gamma (IFN-γ) positive cells early in infection.

Infections caused by parasitic protozoa are very common in humans and animals, and the clinical symptoms may be transient or asymptomatic; but infected, apparently healthy individuals may remain infected throughout life, e.g., toxoplasmosis. Many years of research on *T. gondii* as a model zoonotic pathogen has advanced the field tremendously. Important discoveries continue to be made using this model and provide the basis for translational research. Indeed, findings of articles in this Research Topic show that *Toxoplasma* infection in the cat, the definitive host for *T. gondii*, elicits multi-tissue transcriptional changes mostly associated with immune functions (Cong et al.). Findings by Liu et al. demonstrate that galectin-3 and galectin-9 differentially regulate the expression of microglial M1/M2 macrophage markers and T helper 1/2 (Th1/2) cytokines in the brain of genetically susceptible (C57Bl/6) or resistant (Balb/c) mice, following peroral infection with *T. gondii*. Further, acute infection with *T. gondii* induces M1 polarization in the mouse brain, while chronic infection with the same strain inhibits the harmful Th1-associated inflammatory responses while M1 phenotype is maintained, indicating the complexity of the immune initiation stage upon infection (Hwang et al.). Admittedly, description of macrophage phenotypes and associated functions is rapidly expanding, thereby the intricate role of macrophage lineages in response to *T. gondii* infection warrants extensive investigation.

In North America and Europe, the strains of types I, II, and III *T. gondii* predominate, while in China, type Chinese 1 for *T. gondii* is prevalent. The type Chinese 1 is unique in that it carries a type II GRA15 protein (GRA15II) and a type I ROP16 protein (ROP16I), and known to polarize the host macrophages toward both M1 and M2 phenotypes at the early stage of infection. Using the type Chinese 1 strain deficient of ROP16_I/III_, WH3Δ*rop16*, created with CRISPR/Cas9 technology, it is evident that pregnant mice infected with WH3Δ*rop16* have elevated Th1, but repressed Th2 and Treg responses, leading to pregnancy failure (Wang et al.). This suggests that disturbance of host immunotolerance during pregnancy by *T. gondii* or its sister parasite *Neospora caninum* can contribute to pregnancy failure.

*T. gondii* ROP18 is an essential virulence factor but its interaction with host targets has not been studied extensively. In an effort to identify ROP18 binding partners in human cells, Xia et al. use a high-throughput bimolecular fluorescence complementation (BiFC) approach to reveal numerous human protein partners for the type I and II strain ROP18, most of which are related to immune responses and apoptosis. Future studies will be needed to analyze the physiological significance of such host-parasite protein-based interactions. Further, IFN-γ-induced degradation of tryptophan by indoleamine 2, 3-dioxygenase (IDO) is a crucial anti-*Toxoplasma* mechanism. Recent investigations by Bando et al. show that, of the two IDO isoforms in humans, IDO1 is required for IFN-γ-induced immunity against toxoplasmosis.

Toxoplasmosis is one of the leading foodborne causes of deaths. The disease can be transmitted by oocysts in cat feces and contaminated undercooked meats. While there is a need for an effective vaccine against toxoplasmosis in humans and animals, most vaccines studied thus far are low in protective efficacy and remain at the experimental stage. *Toxoplasma* DNA vaccines containing a single antigen (TgDOC2) and multiple antigens (TgPF, TgROP18, TgROP18, TgMIC6 and TgCDPK3) are reported (Zhang, Elsheikha et al.; Zhang, Hou et al.). Both vaccines achieved considerable protection against challenge in the mouse model of infection. Development of vaccines, particularly efficacious and cost-effective subunit vaccines, is challenging for all parasites. Thus, far, the best protection is still achieved by live attenuated vaccines. Research by Xia et al. reveals that a *T*. gondii mutant (Δldh) lacking lactate dehydrogenases 1 and 2 propagates *in vitro*, but not *in vivo*. Mice vaccinated with this mutant are protected from lethal challenges with wild-type strains of *T. gondii* (Xia et al.). It is very promising that such mutants can provide protection but are unable to replicate in animals, as this may lead to development of live attenuated vaccines and identification of candidate antigens for subunit vaccines. Translational research will be required to assess protective efficacy in target species.

Avian coccidiosis caused by *Eimeria* spp. results in production losses to the poultry industry. Kim, Chaudhari et al. review the literature encompassing many years of research on the complex host responses to this infection that include typical Th1 inflammatory responses and those mediated by Treg cells, highlighting the importance of Th17 T cell subsets in host resistance/susceptibility to coccidiosis. In addition, studies by the same group show that the dietary indoles, ligands for the aryl hydrocarbon receptor, upregulate Treg responses while downregulate Th17 responses, leading to reduced host intestinal lesions caused by coccidiosis (Kim, Lillehoj et al.). This suggests that effective dietary treatments can alleviate coccidiosis-elicited pathogenesis and/or reduce or prevent subsequent opportunistic infections (Kim, Lillehoj et al.).

There are numerous important animal and human pathogens in the phylum Apicomplexa, such as *Plasmodium* spp., *Toxoplasma gondii, Eimeria* spp., *Babesia* spp., and *Cryptosporidium* spp. Natural killer (NK) cells are potent first line effector cells in control of apicomplexan infections by producing IL-12-dependent IFN-γ and direct killing of the parasites and infected cells (Ivanova et al.). However, functions attributed to NK cells may be functions of one of the innate lymphoid cell (ILCs) lineages based on extensive and vigorous research performed since the discovery of ILCs; as such, intensive research is justified to understand the functions of these emerging and important ILCs in the interaction between Apicomplexa parasites and their hosts (Ivanova et al.).

Parasitic helminths are multicellular, eukaryotic, invertebrates of medical and veterinary importance. In the past decades, animal and human parasitic helminth control has relied almost entirely on anthelmintic drugs. However, drug-resistant parasites are emerging, illustrating the need for intense research on the parasitic worms and development of alternative control measures. In addition, prior or existing exposure to, and infection by, helminths can result in polarized immune responses in the host. Such a preexisting immunologic background can unavoidably affect the host immune responses and outcome of infections caused by bacterial, viral or fungal pathogens (Mabbott). Eight articles in this issue, most of which describe original research, emphasize helminths infecting humans and animals. The review article in this Research Topic is focused on helminth-mediated immunoregulation through host pattern recognition receptor (PRR)-dependent and -independent mechanisms (Zakeri et al.), involving host-parasite interactions via parasitic excretory/secretory products and extracellular vesicles. The outcome of such a cross-regulation is two-fold, in general. On the one hand, the worms have evolved to acquire the ability to manipulate the host immune responses in favor of their own survival and parasitism. On the other hand, the host requires exposure to helminth infections for establishing a bystander immunity essential for downregulating deleterious host responses due to allergies and autoimmune diseases. Indeed, the results of recent studies by Ebner et al. indicate that, using a cell line in combination with the infective *Ascaris suum* larvae *in vitro* and analysis by dual-species RNA-Seq, worm exposure elevates host metabolic activities and suppresses some of the chemotactic genes, but does not affect overall immune responsive genes or immune signaling pathways. However, upon contact with the host cell, *Ascaris* larval genes responsible for motor function development and invasion are upregulated, an example of parasitic evasion and promotion of self-development, migration and survival.

*Trichinella spiralis*, an important food-borne pathogen, attenuates collagen-induced arthritis through programmed death 1 (PD1) by downregulating Th1/Th17 responses, an instance of a therapeutic effect exerted by helminth infections (Cheng et al.). Results of the recent studies by these investigators illustrate that infection by *T. spiralis* elevates PD1 in CD4 T cells, and mice infected with the parasite have reduced pathology of collagen-induced arthritis. Moreover, the infection-induced therapeutic effect can be abolished by anti-PD1 antibodies. Lack of such a therapeutic effect is shown in PD1-deficient mice, further demonstrating the importance of PD1 (Cheng et al.). Using a bioinformatics systems approach, Homan et al. further demonstrate the importance of co-infection by epitope networking. In this study, predicted T cell epitopes of multiple helminth species and the associated host responses are analyzed for their ability to bind with MHC molecules, as well as for the presence of T cell-exposed motifs, which are comprised of amino acids in a peptide bound to MHC molecules that engage the T cell receptor. Interestingly, T cell-exposed motifs identified in the study are shared among the helminth species and with the taxonomically unrelated pathogens commonly seen in co-infections. The results indicate that a systems approach must be taken when investigating an infection against a backdrop of co-infection and the potential presence of preexisting immune response bias. In the highly complex process of host-gastrointestinal (GI) parasite interactions, the interrelationship between the parasite and the host GI microbiota should also be considered. *Heligmosomoides polygyrus*, a nematode that infects the small intestine of mice, produces excretory/secretory products with antimicrobial activities (Rausch et al.). Using the germ-free rodent model, research conducted by Rausch et al. shows that the parasite may sense and modulate enteric microbiota, in their favor, by controlled release of nematode-derived antimicrobial excretory/secretory products.

Anti-worm vaccines are considered an important alternative to anthelmintics in the face of rapid emergence of drug resistance in the worm; thus, it is crucial to identify protective vaccine candidates in parasitic worms of interest. The *Haemonchus contortus* serine/threonine-protein phosphatase, an excretory/secretory protein, is identified and characterized for its potential to be a vaccine candidate, based on the protein's ability to modulate cytokine production, immune cell migration, and apoptosis, and to downregulate cell proliferation and MHC protein expression (Ehsan et al.). Immunodominant structural proteins and a wide range of moonlighting proteins are identified using combined 2-dimensional electrophoresis and LS-MS/MS in worm extract as well as surface preparations from the adult tapeworm *Hymenolepis diminuta* (Mlocicki et al.). Some of the proteins may be excellent vaccine candidates. It has been known that granulocytes play a key role in bridging the innate and acquired immunities and mediating clearance of helminth infections. The study by Rajamanickam et al. illustrates that *Strongyloides stercoralis* infection promotes the level of eosinophil, neutrophil, and mast cell granule proteins present in peripheral blood, while anthelmintic drug treatment suppresses them, indicating a direct involvement of activated granulocytes during helminth infection.

Taken together, this Editorial gives a brief overview of the findings presented by all articles in this Research Topic, and we hope that these articles provide readers a cross-sectional view of status of current research on some of the parasites of medical and veterinary importance. This collection of articles provides valuable and timely insights into the need for, and recent developments in, parasite research, the mechanisms underlying infection and pathogenesis, and potential therapeutic and prophylactic interventions. Humans and animals in their life times are confronted with constant threat of infections by, or exposure to, several types of parasites, in addition to a whole battery of other pathogens. Parasitic infections may result in protective immunity for life, biased/compromised immune functions, and/or loss of productivity. As stated earlier, the underlying conditions, such as biased immune status by previous parasitic infection or exposure, can be determining factors influencing potential immunities against pathogens which are irrelevant to those of prior exposure. In the laboratory setting, experiments must be designed in such a way as to yield definitive results. However, in the field, we must appreciate that co-infection of individuals is often inevitable. Again, this Research Topic is focused on host-parasite interactions, and those who contributed to this special issue have addressed the importance and significantly advanced the research in each field. We eagerly look forward to advances in parasitology research in the coming years, and to a greater understanding of the interplay between parasites, the host, and other infections in particular.

## Author Contributions

All authors listed have made a substantial, direct and intellectual contributions to the writing, and approved its publication.

## Conflict of Interest

The authors declare that the research was conducted in the absence of any commercial or financial relationships that could be construed as a potential conflict of interest.

